# Species-Specific Proteins in the Oviducts of Snail Sibling Species: Proteotranscriptomic Study of *Littorina fabalis* and *L. obtusata*

**DOI:** 10.3390/biology10111087

**Published:** 2021-10-22

**Authors:** Arseniy A. Lobov, Irina Y. Babkina, Lavrentii G. Danilov, Alexey E. Masharskiy, Alexander V. Predeus, Natalia A. Mikhailova, Andrei I. Granovitch, Arina L. Maltseva

**Affiliations:** 1 Department of Invertebrate Zoology, St. Petersburg State University, 199034 St. Petersburg, Russia; kriska.irichka@gmail.com (I.Y.B.); a.granovich@spbu.ru (A.I.G.); arina.maltseva@spbu.ru (A.L.M.); 2 Laboratory of Regenerative Biomedicine, Institute of Cytology of the RAS, 194064 St. Petersburg, Russia; 3 Department of Genetics and Biotechnology, St. Petersburg State University, 199034 St. Petersburg, Russia; lavrentydanilov@gmail.com; 4 Core Facility Center, “Development of Molecular and Cell Technologies” and “Biobank”, St. Petersburg State University, 198504 St. Petersburg, Russia; masharsky@biomed.spb.ru; 5 Bioinformatics Institute, 197342 St. Petersburg, Russia; predeus@bioinf.me; 6 Centre of Cell Technologies, Institute of Cytology of the RAS, 194064 St. Petersburg, Russia; natmik@mail.ru

**Keywords:** *Littorina*, mollusca, 2D DIGE, RNA-seq, sibling species, invertebrate reproduction

## Abstract

**Simple Summary:**

Genitalia and reproduction-associated proteins are often species-specific and might evolve rapidly. The situation in which the morphology of the reproductive system is the only difference between two or several closely related species has been reported on multiple occasions. Nevertheless, the reasons for such rapid divergence of the reproductive system is still poorly investigated. To shed some light on the issue, we performed a transcriptomic and proteomic comparison of pallial oviducts from the two sibling species of gastropods *Littorina obtusata* and *L. fabalis*. The main identified differences were associated with three functional groups of genes: transposable elements, which enhance genome variation and promote the evolution of new genes, receptor proteins potentially involved in friend or foe recognition, and various enzymes. We hypothesize that these functional groups reflect both the mechanism (transposable elements) and the directions (friend or foe recognition and reproductive physiology) of the rapid evolution of the reproductive system.

**Abstract:**

Genus *Littorina* subgenus *Neritrema* (Mollusca, Caenogastropoda) includes the “obtusata” group of closely related species (*Littorina obtusata* and *L. fabalis*). The anatomy of the adult reproductive system (pallial oviduct) is the only reliable feature used for species identification in females of these species. Reproductive system anatomy and reproduction-associated proteins often diverge between sibling species. Despite being of high evolutionary interest, the molecular basis of this divergence remains poorly understood. We performed proteotranscriptomic comparison of oviducts of *L. obtusata* and *L. fabalis* by RNA-seq on Illumina HiSeq 2500 and two-dimensional protein electrophoresis (2D DIGE) with MS/MS identification of the species-specific proteins. The interspecies differences in the oviduct were associated with (1) metabolic proteins reflecting overall physiological differences between *L. obtusata* and *L. fabalis*, (2) receptor proteins, and (3) transcripts related to transposable elements (TEs). Various receptors identified may recognize a wide variety of ligands from pathogen-associated molecular patterns to specific carbohydrates on the sperm surface. Therefore, these may participate in immune defense as well as in sperm storage and regulation. Species-specificity of multiple TE sequences (coding for reverse transcriptase and ribonuclease H) may indicate the important role of these genomic elements in the *Littorina* species divergence, which has not been reported previously.

## 1. Introduction

*Littorina obtusata* (Linnaeus 1758) and *L. fabalis* (Turton 1825) are sibling species of the genus *Littorina* subgenus *Neritrema* (Mollusca, Caenogastropoda) which are referred to as the cryptic “obtusata” group [1]. *L. fabalis* and *L. obtusata* sympatrically inhabit the European Northern Atlantic and the Barents Sea’s gravel-stony shores, often together with three species of the “saxatilis” cryptic group (*L. saxatilis*, *L. arcana*, *L. compressa*) [1]. All five species demonstrate species-specific patterns of microbiotope distribution [1,2]. *L. fabalis* is predominately associated with *Fucus serratus*, and *L. obtusata* with *F. vesiculosus* and *Ascophyllum nodosum* (depending on the shore structure and fucoid distribution) [1,3,4]. According to differences in the “host” alga preferences, these species differ in their radula features and dietary preferences [1]. *L. fabalis* and *L. obtusata* are also known to vary significantly in their shell morphology, but the anatomy of the adult reproductive system is the definitive character for species discrimination: number and position of the penial mamilliform glands (PMGs), the penial filament shape in males, and the shape of the bursa copulatrix of the pallial oviduct in females [1]. PMGs are specific glands found in the penises of all species of the *Littorina* genus. These glands have several types of secretory cells, but their exact function is still unknown [1]. The oviduct consists of three parts which have specific functions in egg encapsulation: the albumen gland, capsule gland and jelly gland. The jelly gland also includes a bursa (insemination site), which is connected with a receptacle (sperm storage site) by the sperm groove [1].

The anatomy of the reproductive system and reproduction-associated organs (e.g., gonostylus in Diptera) are often the only changed structures between recently diverged sibling species [5,6]. To quote William G. Eberhard (2010): “Genital evolution requires special explanations because genitalia are often species-specific, and their forms are often more divergent among closely related species than are the forms of other traits such as legs, antennae, and eyes” [5]. Usually, the phenomenon of rapid divergence in reproductive traits is associated with post-copulatory sexual selection. The cryptic female choice (CFC) is a possible manifestation of such selection: females of polyandrous species benefit from being fertilized by conspecific males only; thus, inner mechanisms of prioritization for conspecific sperm function in the female reproductive system would be supported. Similar output may be achieved due to species-specific morphology of copulatory organs, functioning as a key–lock system, or selective mating behavior [5,7,8,9]. Accordingly, copulatory organs of species-specific morphology are not typical for monandrous insect species [5]. CFC might be considered as a combination of behavioural, anatomical, and physiological features that allow a female to control the male contribution to the offspring by the influence of efficiency in the process of insemination (precopulatory CFC) or gamete recognition and fertilization (postcopulatory CFC; e.g., influencing the sperm storage) [8,9].

When rapid divergence of reproductive traits driven by sexual conflict occurs, the divergence of sex-associated genes is also expected. The latter phenomenon was demonstrated for some reproductive proteins from a wide range of invertebrates (see recent reviews [8,10] and references within). The rapid evolution of the reproductive proteins may be driven by the same force as an evolution of morphological traits. Rapidly evolving reproductive proteins have been described in several external fertilizers coexisting in sympatry as well as polyandrous internal fertilizers; although, many exceptions are known, e.g., acrosomal proteins in the sympatric species of the *Diadema* sea urchins are highly conservative, unlike the orthologous proteins in other genera with sympatric species (*Echinometra*, *Heliocidaris*, *Strongylocentrotus*) [8,11,12]. Such examples prompt the extension of the range of models to study the evolution of reproductive traits.

*L. fabalis* and *L. obtusata* represent a pair of cryptic species, diverged very recently (~0.5–1 Mya), most probably in sympatry owing to ecological speciation [4,13,14,15,16]. These species are polyandrous dioecious internal fertilizers still living in sympatry (and accidentally commencing interspecific copulation) [1,4,17]; obviously they need additional mechanisms restricting gene flow. At the same time, there is indirect evidence of conspecific sperm precedence in these species. The unequal contribution of males to progeny was demonstrated for *L. obtusata* and for the phylogenetically close species *L. saxatilis*, which suggests the functioning of CFC in the species of the *Neritrema* subgenus [18,19,20].

The phenomenon of rapid evolution of reproductive proteins in the *Neritrema* species is still rather poorly studied [8]. Earlier we analyzed the divergence of male reproductive proteins between *Neritrema* species (mainly focusing on *L. obtusata*) [21]. We started with the most variable morphological trait and performed a comparative proteomic analysis of PMG. Surprisingly, we revealed no PMG-specific proteins diverged between *L. fabalis* and *L. obtusata* [22]. In contrast, their prostate proteomes included no fewer than 55 species-specific proteins (some of them might not be prostate-specific); at least some of these proteins are expected to be transferred within the seminal fluid to a female during copulation and to affect fertilization success [21]. Additionally, we described a paraspermal protein LOSP (Littorina obtusata sperm protein) in the *Neritrema* snails. Parasperm is specialized non-fertile type of sperm cells which are produced alongside fertile eusperm and also transferred to a female during insemination [23,24,25]. The exact function of this protein is not fully understood yet; however, LOSP demonstrates species-specificity, which is compatible with its involvement in reproductive isolation (e.g., based on CFC) of the *Neritrema* species [21]. It is reasonable to expect that rapidly evolving species-specific proteins of the *Neritrema* seminal fluid interact with their specific counter-components in the female reproductive system [21]. Nevertheless, rapidly evolving reproductive proteins of females of the *Neritrema* species (including *L. fabalis* and *L. obtusata*) were not studied until now. Theoretically, species-specific proteins of the female reproductive system might be also associated with rapidly evolving morphological traits (anatomy of pallial oviducts), additionally contributing to reproductive isolation. In this study, we for the first time performed a proteotranscriptomic comparison of the oviduct of *L. fabalis* and *L. obtusata* to identify some rapidly evolving candidate proteins responsible for isolation between sibling species.

## 2. Materials and Methods

### 2.1. Animals

*L. obtusata* and *L. fabalis* were collected from the sympatric populations of the Barents Sea, Varangerfjord, vicinities of Vadsø (70°04′09.0″ N 30°00′22.3″ E) in 2019 (for transcriptomics analysis) and in the White Sea, Kandalaksha Bay, vicinities of the Moscow State University Biological Station (66°33′08.2″ N 33°06′25.1″ E) in 2019 (for proteomic analysis). The molluscs were transported to the laboratory in thermostatic chambers at +4 °C where they were kept in filtered aerated seawater. For oviduct excision, molluscs were dissected. Species discrimination was performed based on the length of bursa copulatrix relatively to pallial oviduct for females, as described previously [1,2,26]. Excised oviducts were washed and placed into TRIzol (Ambion, Austin, TX, USA) for transcriptome sequencing, or into lysis buffer (4% CHAPS, 2M Thiourea, 7M Urea, 25 mM Tris pH 8.2; Sigma Aldrich, St. Louis, MO, USA) for proteomic analysis. Only the oviducts that were excised with intact receptacles and without visible fragments of the gut were used. For proteomics experiments, the biological materials of six individuals were pooled into one sample, while for transcriptomics, the biological materials of three individuals were pooled into one sample. Two pooled samples (two biological replicates) were prepared for each species for each type of analysis.

All individuals used for the analysis had an approximate age of 2 to 3 years for transcriptomics and of 3 to 4 years for proteomics analysis. All oviducts used had receptacles filled with stored sperm and we assumed that all females were copulated in the season of sample collection.

### 2.2. Transcriptomics Analysis

*RNA isolation* Immediately after preparation, samples were frozen and stored in liquid nitrogen. Before RNA extraction, samples were mechanically homogenized, and RNA was extracted following a standard TRIzol extraction protocol [27]. RNA quality was assessed by capillary electrophoresis using a QIAxcel Advanced System (Qiagen, Hilden, Germany).

*Library preparation and sequencing* Libraries were prepared using an NEBNext Poly(A) mRNA Magnetic Isolation Module and NEBNext Ultra Directional RNA Library Prep Kit for Illumina according to the manufacturer instructions. The samples were sequenced on the HiSeq 2500 platform. Raw transcriptomic data were deposited in NCBI (PRJNA662103).

*Bioinformatics analysis* Transcriptomes were assembled *de novo* using Trinity v2.8.5 in the double-stranded mode with the supertranscripts identification option (“–SS_lib_type RF–include_supertranscripts”) [28]. Assembly quality was assessed using BUSCO v3.0.2 in transcriptome mode (option “-m transcriptome”) with Metazoa OrthoDB v9 collection as a reference set [29]. Expression quantification was done using RSEM v1.3.1 in the stranded paired-end mode (“–strandedness reverse–bowtie2–paired-end”) [30]. Transcripts with TPM (transcripts per kilobase million) less than 1 and length less than 250 were removed.

Annotation of the assembled transcripts was done using eggNOG-mapper V2 [31]. ORF predictions were performed by TransDecoder v 5.5.0 based on protein prediction from Pfam-A and NCBI databases.

### 2.3. Gel-Based Proteomics

*Sample preparation* Samples in lysis buffer were frozen in liquid nitrogen and homogenized in mixer mill Retsch MM 400 (30 Hz, 20 min), then sonicated at 0 °C in an ultrasonic bath for 10 min and stored in ice for 15 min. Finally, the samples were centrifuged for 15 min in 12,000× *g* at 4 °C. The supernatants were aliquoted and stored at −80 °C prior to use.

*Two-Dimensional Difference Gel Electrophoresis* Gel-based proteomics was performed by two-dimensional difference gel electrophoresis (2D DIGE) [32,33]. Prior to electrophoresis, 35 ug of each sample was conjugated with 400 pM of Cy2, Cy3 or Cy5 fluorophores for 2D electrophoresis, according to the manufacturer’s recommendations (Lumiprobe, Moscow, Russia). Subsequently, the samples were mixed together and loaded onto precast IPG-strips for two-dimensional electrophoresis (pH 3–10, 7 cm, BioRad Laboratories, Hercules, CA, USA) by passive rehydration overnight at room temperature in the dark. Each gel contained samples of *L. fabalis* and *L. obtusata* oviduct total lysates and internal standard (mix of all samples included in the analysis). No less than two technical replicates were done for each biological replicate with Cy-dyes swap.

Separation in the first direction was carried out in a Protean IEF Cell (BioRad Laboratories, USA) using the method recommended by the IPG-strip manufacturer: 10,000 Vh, end voltage 4000 V, rapid ramp, 20 °C. After isoelectric focusing, IPG-strips were incubated in two equilibration buffers (6 M urea, 2% SDS, 20% glycerin, 0.375 M Tris, pH 8.8) for 10 min in each. The first with 2% dithiothreitol (Sigma Aldrich, St. Louis, MO, USA) and the second with 2.5% iodoacetamide (Sigma Aldrich, St. Louis, MO, USA).

The second direction of 2D-electrophoresis was performed in an Invitrogen Mini Gel Tank (Thermo Fisher Scientific, Waltham, MA, USA) in precast gels Novex NuPage Zoom 4–12% (NP0330BOX; Thermo Fisher Scientific, Waltham, MA, USA). Multiplex visualization of different Cy-fluorophores was performed with a Typhoon FLA 9500 laser scanner (GE Healthcare, Uppsala, Sweden). Spot matching and statistical analysis were done in PDQuest software (BioRad Laboratories, USA).

The protein spots found in both biological replicates, and at least half of technical replicates for each biological replicate, for only one of the studied species were referred as species-specific.

*Protein identification* All species-specific proteins visible on Coomassie G-250-stained gels were excised and identified by a high-performance liquid chromatography–tandem mass spectrometry (LC–MS) procedure, following the “bottom-up” approach as described earlier [22,26].

The gel fragments were cut to pieces, washed with 50% acetonitrile in 25 mM Tris (pH 8.2) three times, dehydrated with 100% acetonitrile and rehydrated with proteomics grade bovine trypsin solution (20 ng μL^−1^, 25 mM Tris, pH 8.2; Sigma Aldrich, St. Louis, MO, USA) on ice for 60 min. Any excessive trypsin solution was removed, and the gel was covered with 25 mM Tris (pH 8.2). Tryptic digestion was performed at 37 °C overnight. Tryptic peptides were eluted with 50% acetonitrile/0.1% formic acid (Sigma Aldrich, St. Louis, MO, USA) and analyzed using LC–MS (Agilent 1260 coupled with ESI-Q-ToF Agilent 6538, Agilent Technologies, Santa Clara, CA, USA). The gradient elution method was 0% B phase to 60% B phase for 40 min and further to 100% B phase (with corresponding decreasing of A phase) for 10 min, where B phase was 90% acetonitrile with 0.1% formic acid and A was 5% acetonitrile with 0.1% formic acid; the flow rate 316 was 15 μL min^−1^; and the column was Zorbax B-C18 5 μm grain, 80 Å pores, 150 mm × 0.5 mm (Agilent Technologies, Santa Clara, CA, USA).

Protein identification by MS/MS spectra was performed using an Agilent Spectrum Mill MS Proteomics Workbench Rev B.04.00.127 in the mode ‘Identity’ against the UniProt database (UniProtKB, Mollusca, August 2020, 340,252 sequences) and databases constructed based on ORFs predicted from *L. obtusata* and *L. fabalis* oviduct transcriptomes obtained in this research. The precursor mass tolerance was set to 20 ppm. The validation procedure of identified proteins was performed with a minimum protein score of 20 and a peptide false discovery rate (FDR) for validated proteins of 1%.

## 3. Results

### 3.1. Species-Specific Orthogroups in Transcriptomes

In total, 46,098,082 and 56,983,594 clean reads were obtained for two pooled biological replicates of *L. obtusata* and *L. fabalis*, respectively. *De novo* assembly by Trinity produced 99,513 unigenes with a total length of 10,822 bp and an average length of 720.6 bp for *L. obtusata*. For *L. fabalis* we obtained 58,055 unigenes with 11,253 bp total and 680.1 bp average lengths. For further analyses, only transcripts longer than 250 nt were used.

**Figure 1:**
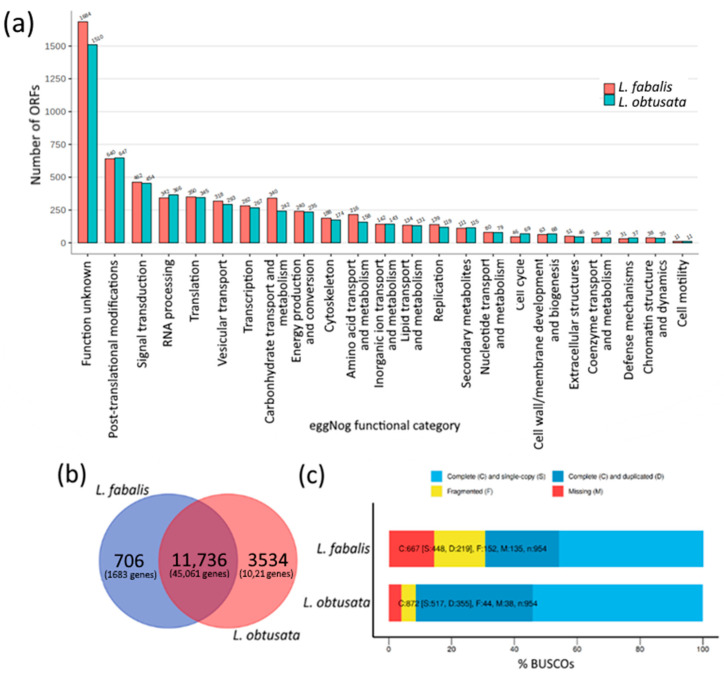
Results of de novo assembly of *L. obtusata* and *L. fabalis* oviduct transcriptomes. (**a**) Number of eggNOG annotated ORFs predicted in *L. obtusata* (blue) and *L. fabalis* (red) oviduct transcriptomes. (**b**) Venn diagram of overlapping and species-specific orthogroups with numbers of genes included in groups. (**c**) BUSCO analysis of *L. obtusata* and *L. fabalis* oviduct transcriptomes. C—complete genes [S—in single-copy, D—duplicated]; F—fragmented genes; M—missing genes; n—total number of genes.

Both obtained assemblies have less than 20% of missed transcripts by BUSCO (Figure 1a). The functional annotation expectedly showed rather similar pattern of protein groups by function in both transcriptomes (Figure 1b). The largest ORF group was assigned to the unknown function; among the top annotated categories were functions related to protein and RNA synthesis and processing, signalling and transport. Concerning the search for rapidly evolving proteins, we performed orthogroup analysis by orthofinder to identify species-specific orthogroups. In total, orthofinder identified 22,609 and 45,544 orthologs for *L. fabalis* and *L. obtusata* respectively, 72.2% (16,320) and 63.1% (28,741) of which were assigned to orthogroups. Most orthogroups and orthologs were shared among species (Figure 1c); 706 and 3532 were species-specific (orthogroups which included unigenes only from one of the species) for *L. fabalis* and *L. obtusata* respectively. For further analysis of the species-specific orthogroups, we used only orthogroups comprising at least three genes: 168 and 1328 unique orthogroups for *L. fabalis* and *L. obtusata*, respectively.

Only a small number of genes from species-specific orthogroups has any known function. Thus, functional annotation by eggNOG-mapper v2 revealed probable functions for only 42 of *L. fabalis* (Table S1) and 1347 of *L. obtusata* (Table S2) species-specific genes, including: (1) proteins associated with transposable elements and viral homologues to reverse transcriptase, ribonuclease H, transposase and K02A2.6; (2) C-type lectins; (3) proteins associated with signal transduction such as homologues of Von Willebrand Factor and MIB2; (4) proteins involved in the metabolism of xenobiotics and stress (cytochrome P450 and heat shock protein 70 family homologues); (5) various enzymes, such as sulfotransferase and metalloaminopeptidase.

### 3.2. Species-Specific Proteins in L. obtusata and L. fabalis Oviducts

**Figure 2:**
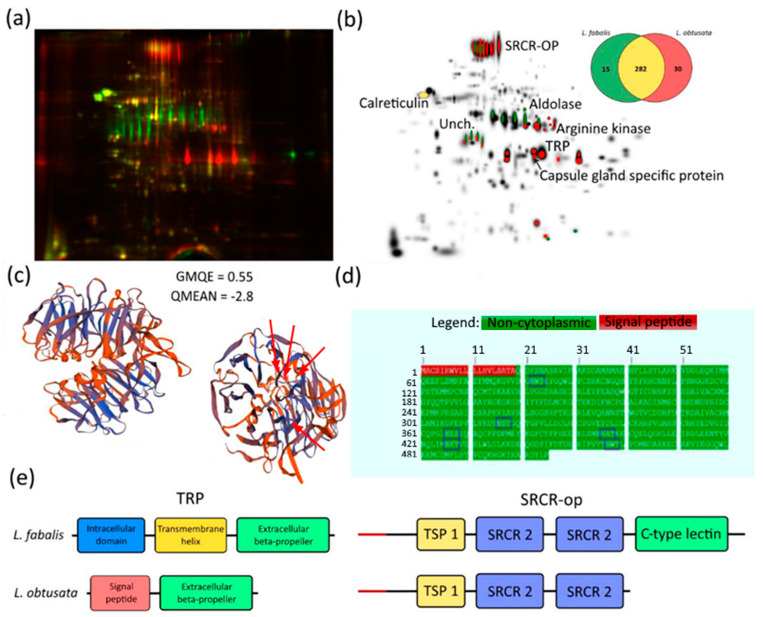
Results of proteomic analysis of *L. obtusata* and *L. fabalis* oviducts. (**a**) 2D DIGE electrophoregram with Cy3 and Cy5 channels merged; green—protein spots of *L. fabalis* oviducts, red—protein spots of *L. obtusata* oviducts. (**b**) PDQuest generated master-gel of all biological and technical replicates; green—*L. fabalis* species-specific spots, red—*L. obtusata* species-specific spots, names—proteins identified by MS/MS and discussed further; Unch.—“uncharacterized protein”; Venn diagram represents numbers of matched and species-specific proteins in proteomes of *L. fabalis* or *L. obtusata* oviducts. (**c**) *Littorina obtusata* Tachylectin-related protein (LoTRP) extracellular domain structure prediction results. The model in Protein Data Bank format provided in the full Western Blot in Supplementary. Protein 3D model was obtained by SWISS-model service [34]; red arrows—species-specific aa substitutions in the beta-sheets of one of the beta-propellers; GMQE and QMEAN Z-scores represent the quality of the obtained model. (**d**) Scavenger receptor cysteine-rich domain-contained oviduct protein (SRCR-OP) structure prediction results by Philius transmembrane prediction service [35]. Blue frames—N-glycosylation sites predicted by NetNGlyc 1.0 Server [36]. (**e**) Block diagram, representing a comparison of the domain structure of TRP and SRCR-OP from *L. obtusata* and *L. fabalis*.

After 2D DIGE, 327 protein spots were included in the comparative analysis. Species-specific proteins spots (SSPS) detected in all biological and technical replicates represented the minor fraction: 15 in *L. fabalis* and 30 in *L. obtusata* (Figure 2b).

The validity of assemblies and ORF prediction was verified by successful MS identification of calreticulin, an abundant conservative housekeeping protein matched in two species based on either UniProt DB or predicted ORFs of both *L. obtusata* and *L. fabalis* oviduct transcriptomes (Table S3, Figure 1).

We identified six SSPS by oviduct transcriptomes. Two species-specific proteins proved to be housekeepers: fructose-bisphosphate aldolase and arginine kinase (Table S3, Figure 2), which agrees with earlier published 2DE data [26].

Two other SSPS present in both species were scavenger receptor cysteine-rich domain-contained oviduct protein (SRCR-OP) and uncharacterized protein (Figure 2b, Table S3).

Both proteins have no significant homologues in the NCBI database. No recognizable domain structure was predicted for the “uncharacterized protein” except for a signal peptide (data not shown). The translated amino acid sequence of the uncharacterized protein was assessed via IUPred3 service for intrinsically disordered protein region prediction, with no disordered regions revealed (iupred.elte.hu; accessed 26 July 2021; data not shown) [37].

SRCR-OP (scavenger receptor cysteine-rich domain-contained oviduct protein) also has a signal peptide; no reliable structural model was obtained by the SwissModel service either. Nevertheless, we identified several conservative domains in the SRCR-OP structure: TSP 1 repeat domain and two SRCR-domains predicted by both InterProScan and NCBI conserved domain database (Figure 2e). While these three domains were present in both species, LfSRCR-OP has an additional C-lectin domain which was absent in its homologue in *L. obtusata*.

The MW and pI of the “uncharacterized protein” calculated by Expasy (web.expasy.org, accessed on 3 August 2021) for the protein without a signal peptide corresponded to those observed in 2D DIGE (Figure 2a), while the empirical MW and pI of SRCR-OP differed from the predicted one. One possible explanation is presence of post-translational modification, e.g., glycosylation of SRCR-OP, which would cause shifts in MW and pI. Accordingly, we found five N-glycosylation sites predicted by the NetNGlyc 1.0 Server (Figure 2d). Finally, there were two *L. fabalis*-specific proteins with known homology in the NCBI databases: tachylectin-related protein (TRP) and capsule gland specific secretory protein (CGSSP) (Table S3, Figure 2).

Although SSPS corresponding to TRP was detected only in *L. obtusata* (Figure 2), TRP-homologous transcript was found in *L. fabalis* as well. Both predicted proteins demonstrate structural homology with horseshoe crab tachylectin. Among several isoforms corresponding to TRP in *L. obtusata* transcriptome, the longest one (including start-, stop-codons and polyA-tail) encodes a 301 aa protein (calculated MW 32.4 kDa and predicted pI 9.04). LoTRP was predicted to include a signal peptide and a globular region (Figure 2e), implying its passage through vesicular compartments. Globular domain demonstrates structural homology to fish-egg lectin (Figure 2c) and consists of two six-blade beta-propellers (also predicted with InterProScan). In *L. fabalis*, LfTRP has a similar six-blade beta-propeller domain (carrying 19 aa substitutions) complemented by an additional transmembrane alpha-helical domain and intracellular domain with no recognizable homologs (Figure 2c–e). Thus, unlike LoTRP, LfTRP appears to be an integral membrane protein.

A protein identified as CGSSP shares homology (70% of identity of translated sequences) with eponymous protein described in the oviduct transcriptome of Japanese marine gastropod *Reishia bronni* (GenBank accession QIQ54707.1). Transcripts corresponding to this protein were found in the transcriptomes of both *L. obtusata* and *L. fabalis* and turned out to be highly conserved—only 4 aa substitutions in the mature proteins were detected, plus a small deletion in a signal peptide region in *L. obtusata*. Theoretical MW/pI for mature protein (without signal peptide) were 21.5 kDa/6.93 for *L. obtusata* and 21.2 kDa/6.93 for *L. fabalis*. Thus, this protein is assumed to have the same localization on the 2D DIGE, but capsule gland specific secretory protein appears to be specific for *L. obtusata* (Figure 2). This inconsistency might come from differences in post-translational modifications or other unknown reasons.

## 4. Discussion

We performed proteomic (2D DIGE) and transcriptomic (RNA-seq with *de novo* assembly) comparison of *L. obtusata* and *L. fabalis* oviducts with the aim at uncovering the molecular background accompanying rapidly evolving morphological traits between sibling species. Most proteins detected via 2D DIGE or predicted based on the transcriptomic data were conserved among the two species. However, a minority of species-specific proteins was also identified based on any of the two applied approaches. The functional groups of the identified differential proteins are discussed below.

*Physiological enzymes* The first functional group includes various types of enzymes involved in general biochemical functions. At the transcriptomic level, the species-specific orthogroups annotated as enzymes were: arylsulfatases, sulfotransferases, methyltransferases, aldo-keto reductases, glycosyl hydrolases, ABC-2 family transporters, aminopeptidases, protein disulfide isomerases, cyclin-dependent kinases, asparaginases, etc. Based on 2D DIGE, only differences in fructose-1,6-bisphosphate aldolase and arginine kinase between species were detected. Robust functional differences between *L. obtusata* and *L. fabalis* were inferred from proteomic and metabolomic screening [17,21]. Our previous proteomic studies in *Littorina* revealed that there are several isoforms of arginine kinase varying between specimens from different microhabitats and between species; some interspecies differences in aldolase features were detected as well [26,38]. Thus, the differences in these enzymes on the protein level are expected and are in good agreement with the previous studies.

Among species-specific orthogroups discovered based on transcriptomic data, only chaperon disulfide isomerase was previously reported to be species-specific [26]. Such low overlap between the results of proteomic and transcriptomics analyses emphasizes complementarity, but not redundancy in these approaches. Gel-based proteomics (2D DIGE) is far less sensitive (only 327 protein spots were analysed), but more effective in the identification of proteoform diversity. In contrast, the transcriptomic approach is much more sensitive (56,957 unigenes identified and included in orthogroups). The drawback of our transcriptomic analysis is related to the differences in the quality of assemblies obtained for *L. obtusata* and *L. fabalis*: at least some orthogroup specificity to *L. obtusata* could be explained by the higher quality of the transcriptomic assembly obtained for this species. Nevertheless, the presence of *L. fabalis* species-specific orthogroups most probably comes from biological differences.

Despite these limitations, structurally diverged orthogroups of two functional categories were identified in the transcriptomes of both species: cytochrome P450 and heat shock protein (HSP) 70 families. Both of these families are associated with stress and their divergence between species might be driven by the ecological differences between *L. obtusata* and *L. fabalis* [1,39,40]. These species tend to be associated with different “host” alga species, to occupy different positions in/on the alga canopy and to pursue different feeding patterns. This determines differential exposition to diverse stressor actions (temperature, desiccation, etc.), as well as toxins, because *L. obtusata* is not only epiphytic but also phytophagous, feeding on the thalli of *Fucus* and *Ascophyllum* and displaying resistance to their toxic polyphenols (unlike *L. fabalis*) [1,17].

*Carbohydrate recognition proteins* Another group with species-specific proteins identified at both proteomic and transcriptomic levels comprises carbohydrate recognition proteins, primarily C-lectins. Three proteins annotated as lectins in the transcriptome of *L. fabalis* and two in that of *L. obtusata* were identified with one protein being species-specific per transcriptome. Another species-specific lectin-like protein was found via the proteomic approach—SRCR-OP. In *L. fabalis,* this protein has the C-type lectin domain which is absent in the ortholog of *L. obtusata*. Although the loss/emergence of the C-lectin domain might be an artefact of *de novo* assembly and should be verified by targeted methods, clear structural differences between two SRCR-OP orthologs were detected via 2D DIGE (Figure 2a).

C-type lectins belong to the variegated superfamily of carbohydrate recognition domain (CRDs)-containing proteins. CRDs of lectins are able to bind carbohydrates and identify them as free sugars or oligosaccharides of glycoproteins and glycolipids. Lectins in metazoans were studied mainly with respect to their participation in the humoral immune response; lectins with immune functions have been widely described in insects, molluscs and other invertebrates [41,42]. Reproductive tracts of invertebrates obviously need to be defended by innate immune mechanisms; moreover, immune effectors may be active players in fertilization success [43]. In the *Littorina* snails, the bursa copulatrix, as well other parts of pallial oviducts studied here, are exposed to potential pathogens due to snails’ copulative activity and are expected to express some immune proteins. Considering the possible dual functionality of the immune effectors in the reproductive system, the revealed differences between lectins of *L. obtusata* and *L. fabalis* may be important not only for strictly immune functions, but for reproductive isolation between siblings as well.

In a similar way, many C-type lectins display other activities besides immune carbohydrate recognition. According to estimates, over 80% of lectins in *C. elegans* and Drosophila have a non-carbohydrate binding function [44]. Non-defensive C-type lectin DDV10, a protein with a C-type lectin domain in the C-terminal, was described in mouse vagina [45], the functional analogue of bursa copulatrix of *Littorina* [1]. Expression of DDV10 is controlled by estrogen and its function is assumed to be associated with the differentiation of epithelial cells [44]. There are also many examples of sperm lectins with functions in reproduction among invertebrates. Bindin, the protein of the sea urchin’s sperm cells surface, is involved in sperm–egg adhesion through binding egg surficial polysaccharides [46]. The putative C-type lectin rAceCTL-1 was described in the hookworm parasite *Ancylostoma ceylanicum*. rAceCTL-1 was identified in sperm and soluble protein extracts of adult males; its presumable function is associated with reproductive physiology [47]. Some lectins may affect sperm storage, such as *D. melanogaster* seminal fluid protein Acp29AB [48]. Listed examples illustrate possible ways of lectin involvement in the regulation of reproduction-related functions, which might be the case for lectins identified in the *Littorina* oviducts.

One more C-type lectin domain-containing protein with a species-specific structure was identified based on 2D DIGE results: SRCR-OP. This putative secreted protein has the thrombospondin 1 (TSP-1) domain, two scavenger receptor cysteine-rich (SRCR) domains, and a C-type lectin domain (recognized in *L. fabalis* only). SRCR is a highly conserved domain that has been found in diverse taxa from mammals to Cnidaria. Similarly to lectins, SRCR-containing proteins often function as innate immune factors, though the whole spectrum of their possible functions is impressively broad. Unlike the structure, none of these functions appear to be universally conserved [49]. Thrombospondins are conserved calcium-binding glycoproteins, most of which can bind various protein partners, often associated with extracellular matrix [50]. Similar to other thrombospondins, based on the prediction of glycosylation sites, SRCR-OP in *Littorina* is also assumed to be a glycoprotein (Figure 2a,d). The exact function of SRCR-OP is to be explored in the future; their involvement in the extracellular matrix organization is quite probable. Importantly, a homologue of *L. obtusata* SRCR-OP is recognizable in the draft transcriptomic assemblies of *L. arcana* testis (pairwise % Identity: 98.2%; data not showed). *L. arcana* SRCR-OP also has a C-type lectin domain, indicating that the presence of the C-type lectin domain is a plesiomorphic state, and its loss represents an evolutionary novelty in *L. obtusata*. In addition, SRCR-OP presence in the male reproductive system suggests that its expression and function are not restricted to the oviduct, although it still may be linked to reproductive physiology.

The tachylectin-related protein (TRP) was found via 2D DIGE in *L. obtusata*. Tachylectins were originally described in circulating hemocytes and hemolymph plasma of the horseshoe crab *Tachypleus tridentatus*, where they function as recognition agents for Gram-negative bacteria lipopolysaccharides [51]. Most other tachylectins are involved in pathogen recognition as well [51,52]. This makes functioning of LoTRP and LfTRP as innate immune effectors quite plausible.

*Retrotransposon-associated proteins* Surprisingly, we discovered several species-specific orthogroups associated with putative transposable elements (TEs): Ribonuclease H (three in *L. fabalis* and seventeen in *L. obtusata*), K02A2.6-like proteins (K02A2.6 is retropepsin-like domain of invertebrate retrotransposons with long terminal repeats; five in *L. fabalis* and seven in *L. obtusata*), as well as reverse transcriptase (three in *L. fabalis* and twentyfive in *L. obtusata*). In *L. obtusata*, several additional TEs elements were present: four transposases, two proteins with integrase core domain, retrotransposable element Tf2 155 kDa protein type 1-like, and six other transposition-associated proteins (The full Western Blot in Supplementary).

Integrase, RNaseH and reverse transcriptase are crucial for retrotransposon replication cycle completion; these elements features are usually used for retrotransposon classification and phylogeny reconstruction [53]. There are no strict differences between retroviruses and retrotransposons; however, there was no recognizable sequences coding for the structural proteins of a virion core and envelope. Therefore, these identified in the *Littorina* oviduct transcriptome sequences should be classified as transcribed TEs. Generally, retrotransposons are common in Mollusca; Thomas-Bulle et al. described 1709 families within 31 LTR retrotransposons within the genomes of 9 mollusc species from 3 classes [53].

Transposons were also recorded in the periwinkle genomes; in particular, Puzakova and Puzakov (2017) compared sequences in the *L. saxatilis* genome with known TEs and identified six sequences similar to the Tc1/mariner DNA transposons [54]. McInerney et al. (2011) performed a comparative genomic analysis of microsatellite containing sequences (MCS) in *L. saxatilis* and *L. littorea* genomes. Similar to our data, they found TEs in both species with significant species-specific differences: *L. saxatilis* had MuDR (MULE) and Mu-like DNA transposons, while *L. littorea* had a variety of TEs: DNA transposons (En/Spm (CACTA), Mariner, hAT, Arnold, MuDR (MULE)); LTR retrotransposon (Gypsy) and non-LTR retrotransposons (LINE and SINEs) [55]. Accordingly, transcripts annotated as transposase were registered in the *Littorina* transcriptomes studied.

Interestingly, we found species-specific transposase and integrase transcripts only in *L. obtusata*, while many other species-specific TEs-associated proteins, such as species-specific proteins with the retropepsin-like domain of invertebrate retrotransposons (K02A2.6-like proteins), were found in both species. One possible reason is the difference in assembly quality of the two species. Nevertheless, transcription of incomplete copies of LTR retrotransposons without segment coding for the reverse transcriptase domain was previously described for *S. mansoni*, and a similar phenomenon may be hypothesized in *L. fabalis* [56].

Summarizing the data discussed above, the species-specific differences in oviduct function are associated with (1) metabolic proteins reflecting overall physiological differences between *L. obtusata* and *L. fabalis* [1,26], (2) receptor proteins, and (3) transcripts related to TEs. Various receptor proteins might be involved in the recognition of a wide range of targets from pathogens to specific carbohydrates on the in the sperm surface and thus to be involved in the regulation of various processes from immune defense to sperm storage. Nevertheless, any screening data are not sufficient to establish the molecular function unambiguously.

The most intriguing difference is the species-specificity of TE-related transcripts. Our data did not allow us to clearly identify the families and the nature of TEs presented in genomes of *L. obtusata* and *L. fabalis*, but we can conclude that some TEs are transcribed in the oviducts of both species. Transcribed retrotransposons are known to participate in gene expression regulation [57], and this may have occurred in the case studied. On the other hand, recently, Gorbushin and Borisova (2015) showed a high level of TE expression in *L. littorea* hemocytes and suggested that transcriptionally active TEs in hemocytes “have an active role in the shaping of genome variation” and promotion of the evolution of new genes [58]. Similarly, transcriptionally active TEs in the oviduct may facilitate in some way the rapid divergence of sibling species at the morpho-anatomical level, as well as in the proteins responsible for the emergence of reproductive barriers [8,21].

Active TEs are known to increase rearrangement rate in adjoining loci [59]. Recently, multiple TEs were recognized in the flanking regions and introns of the gene of the *Littorina* male reproductive protein LOSP (Maltseva et al., submitted). It was suggested, that the insertions/deletions of repetitive motifs in the LOSP structure, determining interspecies variability of this protein, might be related to TEs activity. This, in turn, could be among factors facilitating emergence of reproductive barriers between incipient species during their divergence. The transcriptional interconnection between sex-biased genes and TEs was described in diverse organisms (rev. in [60]). Moreover, non-random distribution of sex-biased genes and TEs was demonstrated in the genome of the medaka fish: these elements form clusters with the same expression bias, and the regulatory links between sexual genes and TEs were inferred [61]. The TEs identified in the *Littorina* oviducts might be expected to be expressed via “hijack” on the transcription of neighboring female-biased genes and to accelerate their evolution. Though being just a hypothesis, this emphasizes the importance of investigation of the interplay between reproductive proteins and TEs, especially in evolutionarily young species.

## 5. Conclusions

We compared pallial oviducts of *L. obtusata* and *L. fabalis* sibling species using proteotranscriptomic analysis. As many as 99,513 and 58,055 unigenes for *L. obtusata* and *L. fabalis* were identified based on RNA-seq *de novo* transcriptome assemblies and 327 proteins by usage of two-dimensional difference gel electrophoresis (2D DIGE). Species-specific proteins and orthogroups were found: 15 proteins/168 orthogroups for *L. fabalis* and 30 proteins/1328 orthogroups for *L. obtusata*.

Functional annotation recognized the minority of species-specific genes with three major functional groups identified by transcriptomics and proteomics. (1) Differences in metabolic proteins reflect overall physiological differences between species. (2) Receptors with non-self-recognition potency may have immune function, as well as be involved in sperm conditioning, storage, etc. (3) Active transposable elements (TEs) such as reverse transcriptase and ribonuclease H, were described for the first time in the female reproductive system of *Littorina*. We hypothesize that TEs accelerate the evolution of genes coding for reproductive proteins, which may be crucial for reproductive barriers emergence. We believe, there are many novel reproductive proteins responsible for interspecific reproductive barriers and post-copulatory sexual selection among not annotated species-specific transcripts, that need to be investigated in details in the future.

## Data Availability

Raw transcriptomic data were deposited in NCBI (PRJNA662103), raw gel-based proteomics data is given in the Supplement to the article.

## Supplementary Materials

The following are available online at https://www.mdpi.com/article/10.3390/biology10111087/s1, Table S1: *L. fabalis*; Table S2: *L. obtusata*; Table S3: *L. obtusata* and *L. fabalis*; Figure S1: Comparison of the results of calreticulin identification using the UniProt database and *L. fabalis* or *L. obtusata* pallial oviduct transcriptomes de novo assembles; Figure S2: Comparison of the results of aldolase and arginine-kinase identification using *L. fabalis* or *L. obtusata* pallial oviduct transcriptomes de novo assembles; Figure S3: Comparison of the results of the scavenger receptor cysteine-rich domain-contained oviduct protein (SRCR-OP) and uncharacterized protein identification using *L. fabalis* or *L. obtusata* pallial oviduct transcriptomes de novo assembles; Figure S4: Comparison of the results of Tachylectin-related protein (TRP) and Capsule gland specific secretory protein identification using *L. fabalis* or *L. obtusata* pallial oviduct transcriptomes de novo assembles.
