# Supplementary material for: Species-Specific Proteins in the Oviducts of Snail Sibling Species: Proteotranscriptomic Study of Littorina fabalis and L. obtusata
Source: Biology (Basel). 2021 Oct 22;10(11):1087. doi: 10.3390/biology10111087 (PMC8614816; doi:10.3390/biology10111087)
Supplement: Supplementary file 1 [file biology-10-01087-s001.zip › Supplementary materials 3.pdf]

Supplementary materials to “Species-specific proteins in the oviducts of snail sibling species: proteotranscriptomic study of *Littorina fabalis* and *L. obtusata*”

Arseniy A. Lobov, Irina Y. Babkina, Lavrentii G. Danilov, Alexey E. Masharskiy, Alexander V. Predeus, Natalia A. Mikhailova, Andrei I. Granovitch and Arina L. Maltseva

Results of MS/MS identification of *L. obtusata* and *L. fabalis* pallial oviduct species-specific proteins found by two-dimensional electrophoresis.

Database	Uniprot	*L. fabalis* oviduct transcriptome	*L. obtusata* oviduct transcriptome			
Protein name	Calreticulin					
Organism	*Littorina littorea*	*Littorina fabalis*	*Littorina obtusata*			
Acccession number	A0A0A7RPS6	TRINITY_DN71817_c0_g1::TRINITY_DN71817_c0_g1_i1::g.4947::m.4947	TRINITY_DN3063_c0_g1::TRINITY_DN3063_c0_g1_i1::g.11774::m.11774			
Score	61,59	34,25	34,25			
Number of distinct peptides	7	4	4			
AA coverage	16,1	12,4	12,4			
MW	48420,3	47618,2	47632,3			
pI	4,44	4,41	4,41			
Similarity with Uniprot sequence	-	97,10%	97,30%			
Similarity with L. fabalis sequence	97,10%	-	99,80%			
List of identified peptides						
Sequence	MW	Number of spectra	Score	Uniprot	*L. fabalis*	*L. obtusata*
(K)AGTIFDNVLITDDADFAK(E)	1925,9491	4	13,74	+	+	+
(R)EKCPRMLLK(R)	1174,6435	1	5,97	+	-	-
(R)FYGISAK(F)	785,4192	2	8,89	+	+	+
(R)FYGLSAK(F)	785,4192	2	5,54	+	-	-
(R)GFDVLQWK(E)	992,52	1	10,13	+	-	-
(K)IDNEKVESGELEADWDFLPPK(K)	2431,1664	1	6,21	+	-	-
(R)LITDNTLEKK(I)	1174,6678	1	7,76	+	+	+
(K)NLLIK(K)	600,4079	1	5,41	-	+	+

Figure S1. Comparison of the results of calreticulin identification using the UniProt database and *L. fabalis* or *L. obtusata* pallial oviduct transcriptomes *de novo* assembles.

fructose-bisphosphate aldolase					
Database	*L. fabalis* oviduct transcriptome	*L. obtusata* oviduct transcriptome			
Acccession number	TRINITY_DN343_c0_g3::TRINITY_DN343_c0_g3_i1::g.11037::m.11037	TRINITY_DN31_c0_g1::TRINITY_DN31_c0_g1_i2::g.5786::m.5786			
Score	46,5	46,5			
Number of distinct peptides	5	5			
AA coverage	10,7	10,1			
MW	39235,9	41231,1			
pI	6,12	6,41			
Similarity in NCBI database					
Percent of similarity	79%	78,51%			
NCBI equence ID	XP_025090601.1				
Protein name	fructose-bisphosphate aldolase-like				
Organism	Pomacea canaliculata (Gastropoda)				
List of identified peptides					
Sequence	MW	Nuber of spectra	Score	*L. fabalis*	*L. obtusata*
(K)ELIDIAR(A)	829,4778	1	8,87	+	+
(K)EQEKELIDIAR(A)	1343,7165	2	6,61	+	+
(K)LFVDILK(E)	847,5288	2	9,62	+	+
(R)LQGIGVENTEENRR(Q)	1614,8194	1	11,11	+	+
(K)NIIPGIK(V)	754,4822	1	10,29	+	+
arginine kinase					
Database	*L. fabalis* oviduct transcriptome	*L. obtusata* oviduct transcriptome			
Acccession number	TRINITY_DN372_c0_g1::TRINITY_DN372_c0_g1_i4::g.11048::m.11048	TRINITY_DN729_c0_g1::TRINITY_DN729_c0_g1_i1::g.8749::m.8749			
Score	54,97	54,97			
Number of distinct peptides	6	6			
AA coverage	16,4	16,4			
MW	39517,2	39455,2			
pI	6,44	6,85			
Similarity in NCBI					
Percent of similarity	82%	82,91%			
NCBI equence ID	AGN95434.1				
Protein name	arginine kinase				
Organism	Semisulcospira libertina				
List of identified peptides					
Sequence	MW	Nuber of spectra	Score	*L. fabalis*	*L. obtusata*
(K)AASGYDDWPTGR(G)	1295,5651	4	16,96	+	+
(K)FGGTLADCIR(S)	1109,5408	1	5,45	+	+
(K)LAATPEFK(A)	876,4825	4	11,87	+	+
(R)LVSAIETMEK(K)	1120,5918	1	6,07	+	+
(R)LVSAIETMEKK(L)	1248,6868	1	5,3	+	+
(K)QLTDDHFLFNDSDRFLK(A)	2111,0192	1	9,32	+	+

Figure S2. Comparison of the results of aldolase and arginine-kinase identification using *L. fabalis* or *L. obtusata* pallial oviduct transcriptomes *de novo* assembles.

SRCR-OP					
Database	*L. fabalis* oviduct transcriptome	*L. obtusata* oviduct transcriptome			
Acccession number	TRINITY_DN97_c0_g1::TRINITY_DN97_c0_g1_i2::g.15903::m.15903	TRINITY_DN24313_c0_g2::TRINITY_DN24313_c0_g2_i1::g.5477::m.5477			
Score	26,11	26,71			
Number of distinct peptides	2	2			
AA coverage	4,9	12,6			
MW	58425,4	26554,7			
pI	5,34	5,99			
List of identified peptides					
Sequence	MW	Nuber of spectra	Score	*L. fabalis*	*L. obtusata*
(K)IMMQEEFLSMR(F)	1414,6527	1	5,56	+	-
(K)KDEQGDYIFDTDRR(I)	1757,8089	8	20,55	+	-
(K)GFEPVLVK(D)	888,5189	1	14,49	-	+
(R)GPVLLDDLHCSGVEYDIDLCK(H)	2418,1316	3	12,22	-	+

Uncharacterized protein					
Database	*L. fabalis* oviduct transcriptome	*L. obtusata* oviduct transcriptome			
Acccession number	TRINITY_DN1386_c0_g1::TRINITY_DN1386_c0_g1_i5::g.2015::m.2015	TRINITY_DN121_c0_g1::TRINITY_DN121_c0_g1_i1::g.17761::m.17761			
Score	25,7	17,69			
Number of distinct peptides	3	2			
AA coverage	11,3	6			
MW	26439,7	26328,4			
pI	5,35	5,23			
List of identified peptides					
Sequence	MW	Nuber of spectra	Score	*L. fabalis*	*L. obtusata*
(R)LLFLK(V)	633,4334	1	8,95	+	+
(K)VNLEADLSFGTK(Q)	1293,6685	1	7,73	+	-
(K)YHPFPPNER(Y)	1156,5534	5	9,02	+	+

Figure S3. Comparison of the results of the scavenger receptor cysteine-rich domain-contained oviduct protein (SRCR-OP) and uncharacterized protein identification using *L. fabalis* or *L. obtusata* pallial oviduct transcriptomes *de novo* assembles.

Tachylectin-related protein					
Database	*L. fabalis* oviduct transcriptome	*L. obtusata* oviduct transcriptome			
Acccession number	TRINITY_DN108_c0_g1::TRINITY_DN10	TRINITY_DN1_c0_g1::TRINITY_DN1_c			
Score	32,57	94,29			
Number of distinct peptides	7	10			
AA coverage	11,3	46,8			
MW	40834,6	29793			
pI	9,76	6,74			
Similarity in NCBI					
Percent of similarity	47,33%	47%			
NCBI equence ID	XP_025110887.1				
Protein name	lectin L6-like				
Organism	Pomacea canaliculata				
List of identified peptides					
Sequence	MW	Nuber of spectra	Score	*L. fabalis*	*L. obtusata*
(K)AIDSYGVIFWGVDDAEK(I)	1884,9014	3	9,1	-	+
(R)EQSFRYPTR(R)	1183,5854	1	6,39	+	+
(R)GSDWIK(I)	705,3566	2	8,47	+	+
(R)GSDWIKIPGDLK(Q)	1328,7209	5	16,66	-	+
(R)KLEYVSSGDGQVLGLDK(W)	1807,9436	1	6,81	-	+
(K)LEYVSSGDGQVLGLDKWGK(I)	2051,0444	1	6,2	-	+
(K)NPSGVAWVQVEGALK(H)	1554,8275	1	7,78	-	+
(K)QVDVGTNIVWGVNSADDIYYR(I)	2384,1517	3	7,78	-	+
(R)VGVSCSNPIGTGWK(I)	1461,7155	4	12,86	-	+
(K)VSGSLSQISAGYAGVWGVNK(N)	1980,0185	2	8,37	+	+

capsule gland specific secretory protein					
Database	*L. fabalis* oviduct transcriptome	*L. obtusata* oviduct transcriptome			
Acccession number	TRINITY_DN2551_c0_g1::TRINITY_DN2551_c0_g1_i1::g.7944::m.7944	TRINITY_DN1759_c0_g1::TRINITY_DN1759_c0_g1_i2::g.3988::m.3988			
Score	23,27	32,9			
Number of distinct peptides	2	3			
AA coverage	10	10,8			
MW	25601,9	27673,3			
pI	8,34	6,97			
Similarity in NCBI					
Percent of similarity	70,35%	68,00%			
NCBI equence ID	QIQ54695.1	QIQ54709.1			
Protein name	capsule gland specific secretory protein				
Organism	Reishia bronni				
List of identified peptides					
Sequence	MW	Nuber of spectra	Score	*L. fabalis*	*L. obtusata*
(R)CVEADYVTVK(V)	1183,5664	1	5,99	+	-
(R)DFPPISVPNVVR(M)	1339,7369	2	17,28	+	-
(R)APLFIPLIDR(V)	1154,6932	2	13,61	-	+
(R)FAPFLDR(C)	865,4567	2	6,76	-	+
(K)LAEVDLPWK(F)	1070,5881	3	9,74	-	+

Figure S4. Comparison of the results of Tachylectin-related protein (TRP) and Capsule gland specific secretory protein identification using *L. fabalis* or *L. obtusata* pallial oviduct transcriptomes *de novo* assembles.
