# Supplementary figures and images for: Species-Specific Proteins in the Oviducts of Snail Sibling Species: Proteotranscriptomic Study of Littorina fabalis and L. obtusata
Source: Biology (Basel). 2021 Oct 22;10(11):1087. doi: 10.3390/biology10111087 (PMC8614816; doi:10.3390/biology10111087)

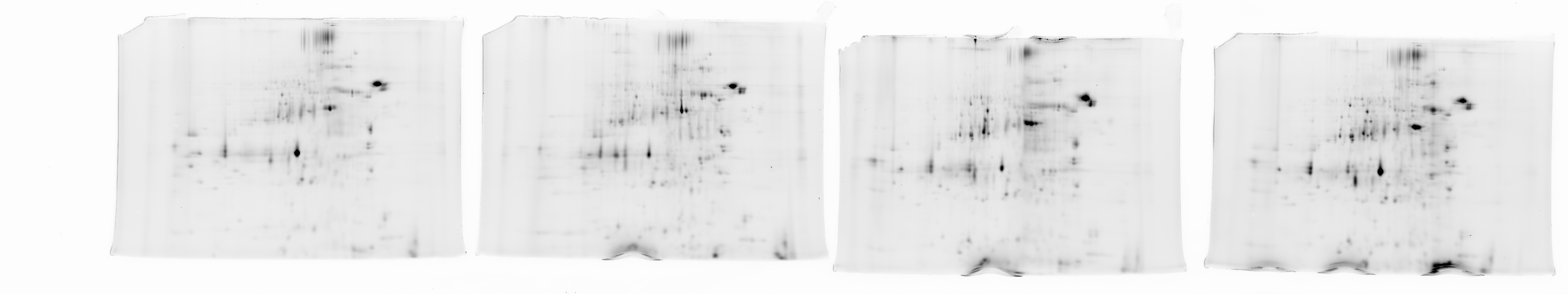

Supplement: Supplementary file 1 [file biology-10-01087-s001.zip › Supplementary materials 4/Ges_1_2_3_4 STANDARD [Alexa Fluor 488].tif]

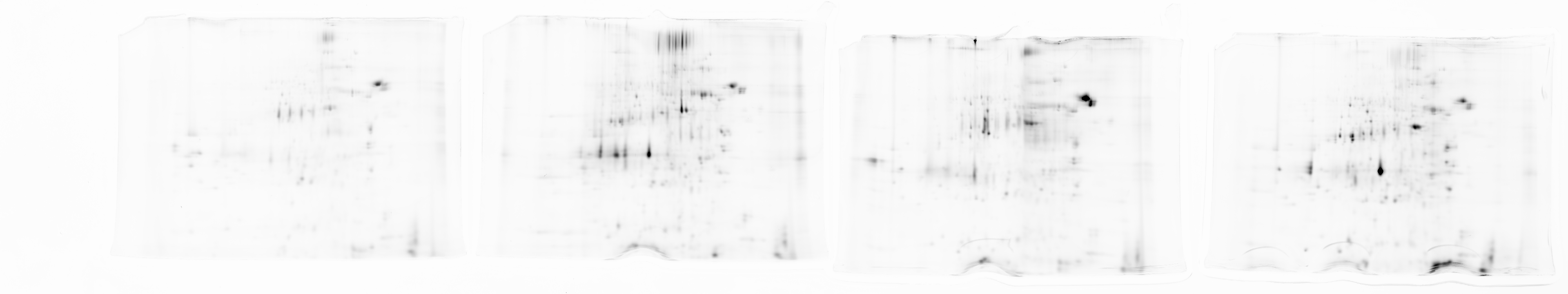

Supplement: Supplementary file 1 [file biology-10-01087-s001.zip › Supplementary materials 4/Ges_1_2_3_4 [Cy3].tif]

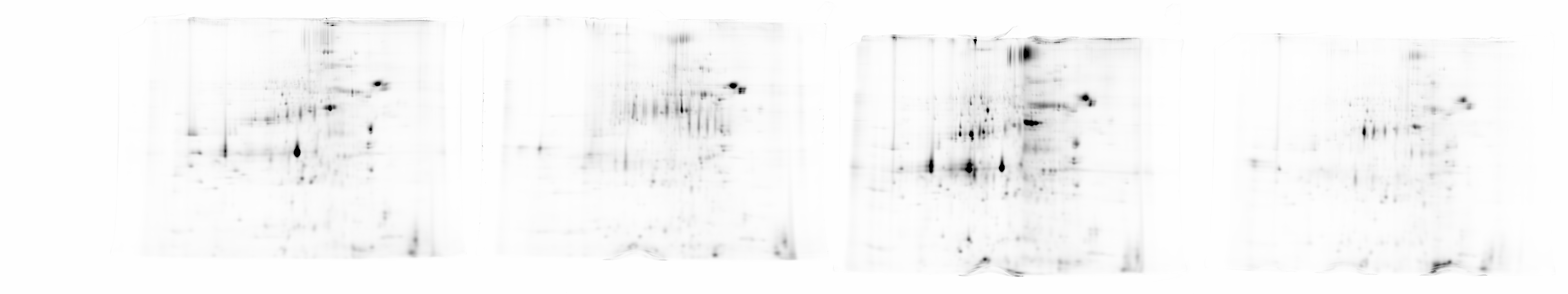

Supplement: Supplementary file 1 [file biology-10-01087-s001.zip › Supplementary materials 4/Ges_1_2_3_4 [Cy5].tif]
